# The cycling brain: menstrual cycle related fluctuations in hippocampal and fronto-striatal activation and connectivity during cognitive tasks

**DOI:** 10.1038/s41386-019-0435-3

**Published:** 2019-06-13

**Authors:** Belinda Pletzer, Ti-Anni Harris, Andrea Scheuringer, Esmeralda Hidalgo-Lopez

**Affiliations:** 1 Department of Psychology, University of Salzburg, Salzburg, Austria; 2 Centre for Cognitive Neuroscience, University of Salzburg, Salzburg, Austria

**Keywords:** Cognitive neuroscience, Sexual behaviour

## Abstract

Estradiol and progesterone vary along the menstrual cycle and exert opposite effects on a variety of neurotransmitter systems. However, few studies have addressed menstrual cycle-dependent changes in the brain. In the present study we investigate menstrual cycle changes in brain activation and connectivity patterns underlying cognition. Thirty-six naturally cycling women underwent functional MRI during two cognitive tasks: spatial navigation and verbal fluency. While no significant performance differences were observed along the menstrual cycle, the changes in brain activation patterns are strikingly similar during both tasks. Irrespective of the task, estradiol boosts hippocampal activation during the pre-ovulatory cycle phase and progesterone boosts fronto-striatal activation during the luteal cycle phase. Connectivity analyses suggest that the increase in right-hemispheric frontal activation is the result of inter-hemispheric decoupling and is involved in the down-regulation of hippocampal activation.

## Introduction

Estradiol and progesterone levels fluctuate along the female menstrual cycle [1]. While both hormones are low during menses, estradiol peaks in the pre-ovulatory phase to drop again and rise to medium levels in the mid-luteal phase. Progesterone is generally low prior to ovulation and rises to peak levels in the mid-luteal phase. Neuroactive effects of sex hormones have received increasing attention and have been ascribed a modulatory role in cognitive functions [2, 3]. However, studies on menstrual cycle-dependent changes in cognition have yielded inconsistent results [4–6]. While some studies report improved verbal, but impaired visuo-spatial abilities during the luteal phase [7–9], others do not confirm these results [10–13]. Thus, recent reviews conclude that menstrual cycle dependent changes in cognitive functions are small compared to more pronounced emotional changes [4]. However, neuroimaging studies suggest that not all neuroactive effects of sex hormones are immediately reflected at the performance level [14–20]. Specifically, if compensatory mechanisms are at play, no changes in performance are expected, while changes in brain activation and connectivity could shed light on the underlying neuronal circuitry supporting task performance.

There are two methodological issues that may have contributed to the current inconsistencies. First, most behavioural studies focus on changes in rather coarse overall performance measures. However, menstrual cycle dependent-changes in cognition are likely more subtle and possibly not so much reflected in performance per se, but in how that performance is achieved, i.e. in cognitive strategies. First evidence supporting this assumption comes from menstrual cycle studies on spatial navigation, reporting that during their luteal phase women focus more strongly on landmark-information and navigate more easily from an ego-centric perspective rather than an allocentric perspective [21, 22]. Accordingly, studies on menstrual cycle dependent changes in cognition should take into account potential shifts in cognitive strategies that might account for their findings.

Second, most human menstrual cycle studies compare menses (low hormone) and mid-luteal phase (medium estradiol, high progesterone), following the rationale that women should behave more male-like, when their female sex hormones are low, and more female-like when their female sex hormones are high [7]. Likewise, the small number of neuroimaging studies in this area have mostly compared menses to either the pre-ovulatory [14, 23, 24] or the mid-luteal phase [12, 16, 17, 22], but only one study so far has included all three cycle phases [20]. They mostly report increased activation in higher hormone phases, but so far there is little consistency with respect to the affected areas. For instance menstrual cycle changes were observed in the parietal and frontal lobes during visuo-spatial tasks [12, 14], inferior frontal gyrus during verbal memory tasks [14–16] and medial prefrontal cortex during numerical processing [17]. Furthermore, changes in the anterior cingulate cortex (ACC) were observed during response inhibition [18–20] and changes in the dorsolateral prefrontal cortex (DLPFC), hippocampus and striatum during working memory [23, 24]. The problem with this approach is the assumption, that estadiol and progesterone have similar effects and hypothesise impaired spatial but improved verbal performance during the mid-luteal phase [4]. This rationale stands in contrast to sex hormones interactions with neurotransmitter systems [2]. While estradiol enhances glutamatergic neurotransmission and reduces GABA-ergic neurotransmission (excitatory effects), progesterone reduces glutamatergic neurotramsnission and enhances GABA-ergic neurotransmission (inhibitory effect). Opposite effects of both hormones on dopaminergic neurotransmission are also suspected [2]. Accordingly, during the luteal phase, when estradiol levels are moderate and progesterone levels high, their effects might cancel each other out, in which case we wouldn’t expect performance differences compared to menses. In order to disentangle the effects of estradiol and progesterone along the menstrual cycle, it is necessary to first look at the effects of estradiol alone during the pre-ovulatory phase. In a second step we can then assess whether progesterone enhances or counteracts the effects of estradiol by comparing the mid-luteal phase to the pre-ovulatory phase.

Animal studies of similar design have suggested beneficial effects of estradiol on spatial working memory [25–27]. These results contrast the findings from human menstrual cycle studies, which have argued for detrimental effects of estradiol on spatial performance [7, 8, 28]. It has been speculated that this improvement in cognitive performance results from an estradiol-dependent increase in hippocampal spine density [3, 29]. Animal studies have also demonstrated an estradiol-dependent increase in spine density in the prefrontal cortex [30]. An increase in hippocampal grey matter has also been reported in humans [31–34]. While results regarding grey matter changes in the prefrontal cortex are inconsistent in humans, the DLPFC shows increased activation during high hormone phases in working memory tasks [24, 35]. These studies included only two menstrual cycle phases focusing on estradiol, but not progesterone. In a follow-up study, we also observed increased activation in the right DLPFC during verbal working memory in the luteal phase [unpublished data].

Furthermore, human menstrual cycle studies repeatedly demonstrate a progesterone-dependent increase in grey matter volumes of the right basal ganglia during the luteal phase [31, 34]. The basal ganglia also show menstrual cycle-dependent changes in brain activation. Specifically, during the luteal cycle phase, the putamen shows increased activation during working memory [unpublished data], while the caudate shows increased fluctuatory activity in the resting state [unpublished data].

We argue that inconsistent results regarding cognitive changes along the menstrual cycle in human studies, may be due to a flawed rationale. Here we address the issue from a different view-point, integrating previous results from the animal and human literature. We hypothesize an estradiol-dependent improvement in cognitive performance, specifically spatial performance. We also hypothesize that this improvement is due to an estradiol-dependent increase in activation of the hippocampus and/or DLPFC, as animal studies demonstrate an estradiol-dependent increase in spine density in the hippocampus and prefrontal cortex [29, 30, 36]. Furthermore, based on previous human studies, we expect a progesterone-dependent increase in activation of the basal ganglia (putamen, caudate) during the luteal phase [31, 34] and explore its relationship to cognitive performance. In addition, we explore potential strategy-related changes in performance and brain activation.

To that end, 36 naturally cycling women were scanned three times along their menstrual cycle to assess their performance and blood oxygen level dependent (BOLD)-response in specific areas of interest while performing a spatial navigation and a verbal fluency task. One session was scheduled during menses, when both estradiol and progesterone levels are low, one during the pre-ovulatory estradiol peak and one during the mid-luteal phase, when progesterone levels are high. Based on previous findings, areas of interest for both tasks include (i) the hippocampus, (ii) the basal ganglia (putamen, caudate), and (iii) the DLPFC.

## Methods

### Participants

Thirty-seven women were scanned three times across their menstrual cycle. One subject was excluded because neither estradiol nor progesterone levels were in accordance with the subject’s self-reported cycle phases (see Hormone analysis). Accordingly, data of 36 women were analysed with a mean age of 25.36 years (SD = 4.42), a mean IQ of 110.89 (SD = 9.61) and a mean cycle duration of 28.83 days (SD = 2.58). Participants had no history of psychological, endocrinological or neurological illness and showed no brain tissue abnormalities on the structural MRI.

### Ethics statement

All participants gave their informed written consent to participate in this study. The study was approved by the University of Salzburg’s ethics committee. The methods conform to the Code of Ethics of the World Medical Association (Declaration of Helsinki).

### Procedure

During a pretest, participants signed the informed written consent, completed a health screening questionnaire, as well as the Advanced Progressive Matrices by Raven [37] to assess their IQ. They were then trained in the cognitive tasks employed during the fMRI scanning sessions. Each scanning session included a resting-state functional scan, a task-based functional scan, a high-resolution structural scan, and a diffusion weighted scan (see fMRI data acquisition). This manuscript focuses on results from the task-based functional scan. Scanning sessions were scheduled during (i) menses (average cycle day ± SD: 3.56 ± 1.50), (ii) the pre-ovulatory estradiol peak (average cycle day ± SD: 12.42 ± 2.77) and (iii) the mid-luteal phase, when progesterone levels peak (average cycle day ± SD: 22.11 ± 3.93). The order of cycle phases was counterbalanced across scanning sessions. Cycle phases were determined as follows: Menses sessions were scheduled two to six days after the onset of menses. Based on participant’s self-reports of their last three menstruation onset dates, an average cycle duration was calculated. The expected onset of next menses was determined by adding the average cycle duration to the onset of last menses. The expected ovulation date was determined by subtracting 14 days from the expected onset of next menses. Pre-ovulatory sessions were scheduled 2–3 days before the expected ovulation and confirmed by commercial ovulation tests, measuring the LH-surge (Pregnafix©). Luteal sessions were scheduled 3–10 days after ovulation as confirmed by ovulation tests and were further confirmed by the onset of next menses.

### Hormone analysis

Participants were instructed not to eat or drink for about 1 h before testing. During each scanning session, participants gave three saliva samples, one upon arrival at the lab after rinsing their mouth with water, one immediately before scanning and one after scanning. Samples were collected via unstimulated passive drool method using 15 ml Greiner tubes. Saliva samples were immediately frozen and stored at −20° until analysis. To remove particulate matter prior to analysis, saliva samples were centrifuged twice for 15 and 10 min respectively at 3000 rpm in an Eppendorf 3750 centrifuge. Saliva from the three samples was pooled prior to analyses to account for fluctuation in hormone release and saliva production. Pooling has the advantage of providing a more stable assessment for the average hormone levels throughout the experiment. Estradiol was assessed using the high sensitivity (HS) Estradiol in Saliva ELISA by Salimetrics with a sensitivity of 1 pg/ml. Progesterone was assessed using the Progesterone in Saliva ELISA by DeMediTec with a sensitivity of 10 pg/ml. All samples were assessed in duplicates and assessment of samples with more than 25% variation between duplicates was repeated. We expected participants to show higher estradiol levels during pre-ovulatory phase compared to menses and higher progesterone levels during luteal phase compared to menses. One participant was excluded, because neither criterion was met.

### Cognitive tasks

In order to diversify cognitive testing for participants, navigation items were alternated with verbal fluency items. Stimuli were created and presented using the Unreal Engine 4 Version 8.1. Items were presented in a block-design for 30 s, followed by 15 s inter-stimulus-intervals. Three versions of the tasks were counterbalanced over scanning sessions and cycle phases.

#### Navigation

The navigation task was adapted from previous 2D and 3D versions developed for behavioural testing [21, 38]. Participants were placed in a virtual environment represented by a 10 × 10 matrix with one of 10 landmarks (house, church, tree, stone, bench, stairs, traffic lights, flowers, bushes) placed on each field. Each landmark occurred only once in each row and column. Participants received directions to a target location. Once they reached that target location, new directions appeared on the screen to a new target location and so on. Their task was to reach as many target locations as possible within 30 s. Previous results [21, 38] demonstrated that navigation performance is influenced by framing directions from different perspectives (allocentric vs. egocentric) and using different strategies (landmark-based vs. Euclidian-based). The allocentric perspective refers to locations in the environment via cardinal directions (“north”, “south”, “west”, “east”). The egocentric perspective places them relative to one’s own body position (“left”, “right”, “straight ahead”). A landmark-based strategy uses landmarks in the environment (e.g. “house”, “tree”, “bridge”) as orientation points. A Euclidian strategy uses absolute distances. In order to address potential strategy shifts along the menstrual cycle, perspective and strategy were varied among directions in a 2 × 2 design: (i) allocentric Euclidian (e.g. “go 4 fields to the north”), (ii) allocentric landmark-based (e.g. “go north until you reach the tree”), (iii) egocentric Euclidian (e.g. “go 4 fields to the left”) and (iv) egocentric landmark-based (e.g. “go left until you reach the tree”). The order of conditions was pseudo-randomised, i.e. each condition was equally often followed by each other condition.

#### Verbal fluency

In the verbal fluency task participants were presented with a semantic category (e.g. farm animals) and were asked to covertly (in order to avoid movement artefacts) produce as many words as possible belonging to that category. Two strategies have previously been described for verbal fluency: clustering and switching [39]. Clustering refers to the more automatic process of producing consecutive words from the same subcategory. Switching refers to the more controlled process of producing consecutive words from a variety of different subcategories, usually resulting in a larger number of words produced overall [39]. In order to address potential shifts between these strategies along the menstrual cycle, clustering and switching conditions were alternated. In the clustering condition, participants were asked to make sure that consecutive words belonged to the same sub-category (e.g. birds) and to switch to a different subcategory only if no other words belonging to the first sub-category came to mind. In the switching condition, participants were asked to make sure, that consecutive words did not belong to the same sub-category. To obtain an indicator of performance, participants were asked to press a button for each word that came to mind. They were instructed to switch buttons, whenever switching sub-categories. One subject did not respond by button presses and was excluded from analyses on the verbal fluency task. In the pretest they said the words that came to mind aloud and their responses were recorded. As an additional control, after each scanning session, participants were asked to reproduce the words for a random category. Categories were pre-screened for overall difficulty (number of words produced if no specific instruction is given), clustering difficulty (number of words produced under the clustering instruction) and switching difficulty (number of words produced under the switching instruction) in a sample of 45 men and 45 women. Difficulty was counterbalanced across versions and categories.

### fMRI data acquisition

Whole-brain fMRI data were acquired on a Siemens Magnetom Trio Tim 3-Tesla scanner at the Christian Doppler Klinik (Salzburg, Austria). The task-based functional scan comprised a T2*-weighted gradient echo planar (EPI) sequence sensitive to BOLD contrast (TR = 2250 ms, TE = 30 ms, FOV 192 mm, matrix size 192 × 192, slice thickness = 3.0 mm, flip angle 70°, voxel size 3.0 × 3.0 × 3.0 mm, 36 transversal slices parallel to the AC-PC line). High-resolution structural images were acquired using a T1-weighted sagittal 3D MPRAGE sequence (TR = 2300 ms, TE = 2.91 ms, TI delay of 900 ms, FOV 256 mm, slice thickness = 1.00 mm, flip angle 9°, voxel size 1.0 × 1.0 × 1.0 mm, 160 sagittal slices).

### fMRI data analysis

During pre-processing, the first 6 images of each scanning session were discarded and the remaining scans were despiked using 3d-despiking as implemented in AFNI (afni.nimh.nih.gov). Images were realigned and unwrapped using Statistical Parametric Mapping software (SPM12) and six movement parameters were extracted. For the identification and correction of non-physiological noise a biophysically-based model (Functional Image Artefact Correction Heuristic, FIACH [40]) was applied. Images were filtered and six parameters of physiological noise were extracted via principal components analyses from a time-series signal-to-noise ratio (TSNR) map. The filtered images were then subjected to the SPM12 standard pre-processing pipeline including slice-timing, co-registration of functional to structural images, segmentation of structural images using the computational anatomy toolbox for SPM (CAT12) and normalization of functional images using the parameters obtained by CAT12. Finally, data were resampled to isotropic 3 × 3 × 3 mm voxels and smoothed with a Gaussian kernel of 6 mm.

A two-stage mixed-effects model was applied for statistical analysis. In the subject-dependent fixed-effects first-level analysis, six regressors of interest were modelled separately to predict BOLD responses to the different types of events: (i) allocentric Euclidian navigation, (ii) allocentric landmark-based navigation, (iii) egocentric Euclidian navigation, (iv) egocentric landmark-based navigation, (v) verbal fluency with clustering instruction, (vi) verbal fluency with switching instruction. Episodes during which instructions appeared on the screen, as well as the six realignment parameters and the six physiological noise parameters obtained from the FIACH procedure were entered as regressors of no interest to the models. All regressors were obtained by convolving the duration of the event with the canonical hemodynamic response function implemented in SPM. A high pass filter cut-off was set at 128 s and autocorrelation correction was performed using an autoregressive AR(1) model [41].

At the first level, we defined one statistical contrast over each regressor of interest, i.e. one for each task condition. Contrasts were then scaled [42] by dividing the contrast image by the ALFF (amplitude of low frequency oscillations) map [43] obtained from estimation residuals using the Data Processing for Analysis of Brain Imaging (DPABI) toolbox [44] and a band-pass filter of 0.01–0.08 Hz. The scaled contrast images from each subject, session and condition entered separate one-sample *t*-test for navigation and verbal fluency at the second level. These models were then used to extract eigenvalues as measures of BOLD-response from a series of regions of interest (ROIs) that were defined using masks based on Brodman-areas (BA) as implemented in the Wake Forest University (WFU) Pickatlas toolbox [45]. Bilateral ROIs for both tasks were: (i) hippocampus, (ii) putamen, (iii) caudate and (iv) DLPFC (BA 46). An additional ROI for navigation was the parahippocampus (PH, BA 36). An additional ROI for verbal fluency was the bilateral inferior frontal gyrus (IFG, BA 44/45).

### Connectivity analyses

Connectivity analyses using bilateral hippocampus, caudate and DLPFC as seeds were performed for both tasks using the CONN-toolbox [46]. The pre-processed functional images underwent linear detrending for white matter (WM) and cerebrospinal fluid (CSF) influences, a band-pass filter (0.008–0.09 Hz) and motion-correction. ROI-to-ROI connectivity analyses were used to extract *Z*-scores for (i) inter-hemispheric connectivity between the bilateral DLPFC and (ii) connectivity between the right DLPFC and subcortical areas.

### Statistical analysis

Statistical analysis was carried out in R 3.4.0. To compare sex hormone levels, performance, brain activation (eigenvalues from ROIs) and connectivity (*Z*-scores between ROIs) across cycle phases, we performed linear mixed-effects models (LMEs) using the *lme* function of the *nlme* package [47]. Cycle phase was the main factor of interest in these analyses. Power simulations for LMEs were run using the *longpower* package [48]. The power for detecting a cycle effect, while controlling for session and task-conditions (see below) in a sample of 36 participants is 99.5%. A first model included all three cycle phases and compared the high hormone phases (pre-ovulatory, luteal) to menses. In a second model the difference between pre-ovulatory and luteal phase was evaluated. Even though session was counterbalanced across cycle phases, we included session as a factor for all LMEs, in order to control for potential learning effects. For all models on the navigation task, perspective and strategy were additionally included as factors to control for task-based effects. For the same reason, condition was included as a factor in all models on the verbal fluency task. An interaction between cycle*condition was considered as indicative for a strategy shift along the menstrual cycle. If no cycle*condition interactions were observed, they were removed from the respective models. Particularly no cycle*condition interactions were observed for performance or brain activation. Accordingly, the interaction terms perspective*cycle, strategy*cycle or condition*cycle were removed from the respective models. For all models, session and strategy effects are reported in Supplementary Material 1. In order to address, whether significant menstrual cycle effects were attributable to estradiol or progesterone levels, models yielding a significant menstrual cycle effect were re-run replacing the factor *cycle* by estradiol, progesterone and their interaction. All continuous dependent and independent variables were scaled prior to analyses in order to allow an interpretation of effect sizes based on standard deviations (similar to Cohen’s d). For each effect of interest, p-values were FDR-corrected for the 10 bilateral ROIs assessed for each task. Data and scripts are openly available at http://webapps.ccns.sbg.ac.at/OpenData/. MR-images are available upon request from the first author.

## Results

### Hormones

**Fig. 1:**
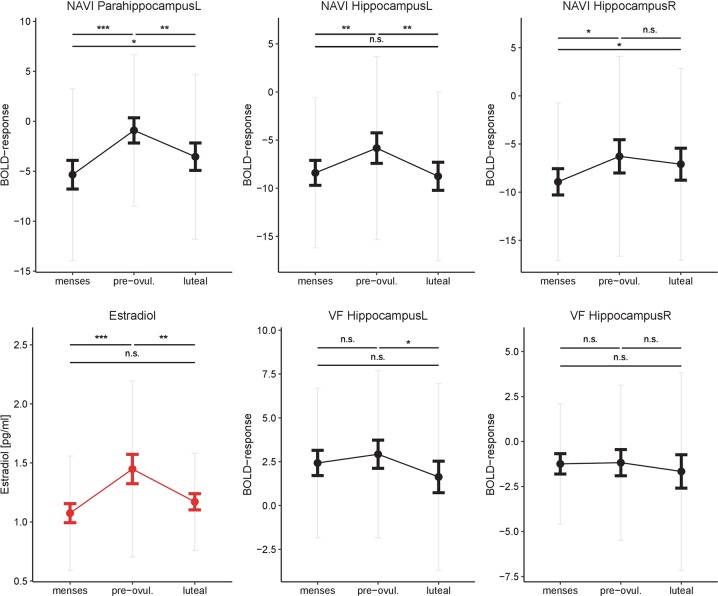
Changes in estradiol and hippocampal activation along the menstrual cycle. Estradiol and hippocampal activation during both tasks were significantly increased during the pre-ovulatory phase of the menstrual cycle. For navigation, the same pattern was observed in the parahippocampus (only left parahippocampus shown). Bold error bars represent standard errors. Grey error bars represent standard deviations. NAVI navigation, VF verbal fluency, L  left, R  right. *p < 0.05, **p < 0.01, ***p < 0.001, n.s. not significant

Estradiol (Fig. 1) was significantly higher during the pre-ovulatory phase (1.45 pg/ml, SD = 0.74 pg/ml) compared to menses (1.07 pg/ml, SD = 0.48 pg/ml; *b* = 0.64, SE_b_ = 0.14, *t*_(70)_ = 4.50, *p* < 0.001) and luteal phase (1.17 pg/ml, SD = 0.41 pg/ml; *b* = −0.45, SE_b_ = 0.17, *t*_(35)_ = −2.67, *p* = 0.01), but did not differ between luteal phase and menses (*b* = 0.17, SE_b_ = 0.14, *t*_(70)_ = 1.16, *p* = 0.25).

**Fig. 2:**
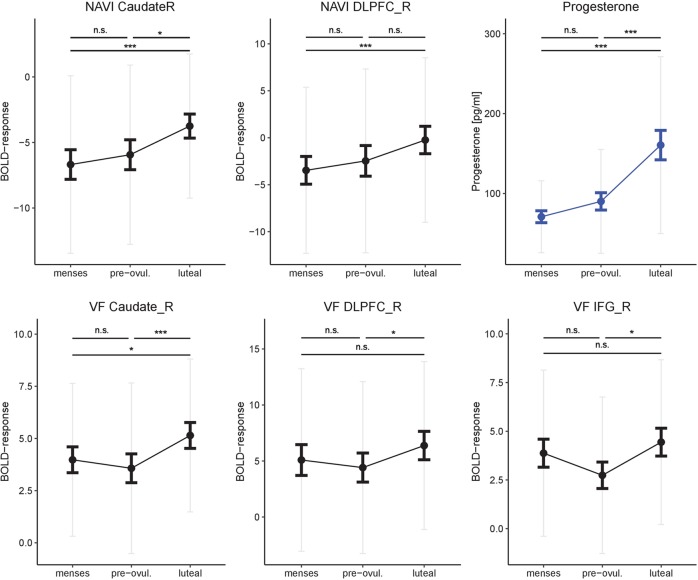
Changes in progesterone, striatal and prefrontal activation along the menstrual cycle. Progesterone, striatal and right dorsolateral prefrontal (DLPFC) activation increased significantly during the luteal phase of the menstrual cycle. In the verbal fluency task, the same pattern was observed in the right inferior frontal gyrus (IFG; only left IFG shown). Bold error bars represent standard errors. Grey error bars represent standard deviations. NAVI navigation, VF verbal fluency, L left, R right. *p < 0.05, **p < 0.01, ***p < 0.001, n.s. not significant

Progesterone (Fig. 2) was significantly higher during the luteal phase (160.62 pg/ml, SD = 110.72 pg/ml) compared to menses (70.92 pg/ml, SD = 44.93 pg/ml; *b* = 1.03, SE_b_ = 0.16, *t*_(70)_ = 6.37, *p* < 0.001) and pre-ovulatory phase (90.14 pg/ml, SD = 65.07 pg/ml; *b* = 0.73, SE_b_ = 0.18, *t*_(35)_ = 4.07, *p* < 0.001), but did not differ between pre-ovulatory phase and menses (*b* = 0.22, SE_b_ = 0.16, *t*_(70)_ = 1.37, *p* = 0.18).

### Performance

In both tasks, menstrual cycle effects on performance did not reach significance after controlling for session (all *b* < 0.08, all SE_b_ > 0.08, all *t* < 0.90, all *p* > 0.35).

### Brain activation

Effect sizes and standard errors for menstrual cycle effects are summarised in Supplementary Table 1. *T* and *p* values are reported in the text. In both tasks, no cycle-dependent changes in BOLD-response were observed in the bilateral putamen and left DLPFC (all *t* < 1.70, all *p* > 0.08).

#### Navigation

Activation in the bilateral hippocampus and parahippocampus (Fig. 1) was significantly increased during the pre-ovulatory phase compared to menses (all *t* > 2.62, all *p*_FDR_ < 0.02). In the left hemisphere this activation decreased again in the luteal phase (both *t* < −2.69, both *p*_FDR_ < 0.03), while this was not the case in the right hemisphere (both |*t*| < 0.75, both *p*_FDR_ > 0.76). Hippocampal activation was bilaterally related to estradiol (both *t* > 2.81, both *p*_FDR_ < 0.02) and a significant estradiol*progesterone interaction was observed (both *t* < −3.17, both *p*_FDR_ < 0.002). Likewise, left parahippocampal activation was related to estradiol levels (*t* = 2.40, *p*_FDR_ = 0.04) and a significant estradiol*progesterone interaction was observed (*t* = −2.67, *p*_FDR_ = 0.009).

Activation in the bilateral caudate and right DLPFC (Fig. 2) was significantly increased during the luteal phase compared to menses (both *t* > 3.83, both *p*_FDR_ < 0.001). Accordingly, activation in the right caudate and DLPFC were significantly related to progesterone (both *t* > 3.27, both *p*_FDR_ = 0.004) and a significant estradiol*progesterone interaction was observed (both *t* < −3.23, both *p*_FDR_ < 0.002).

#### Verbal fluency

Activation in the left hippocampus (Fig. 1) did not differ between the high hormone phases and menses (both |*t*| < 1.34, both *p* > 0.18), but was significantly reduced in the luteal phase compared to the pre-ovulatory phase of the menstrual cycle (*t* = −2.05, *p*_FDR_ < 0.05). We observed no significant association to estradiol (*t* = 2.39, *p*_FDR_ = 0.10), but a significant estradiol*progesterone interaction (*t* = −3.17, *p*_FDR_ = 0.01). No menstrual cycle dependent changes in activation were observed in the right hippocampus.

Activation in the bilateral caudate and the right DLPFC (Fig. 2) did not differ between pre-ovulatory phase and menses (all |*t*| < 1.10, all *p* > 0.40), but was significantly increased during the luteal phase compared to the pre-ovulatory phase (all *t* > 2.85, all *p*_FDR_ < 0.02). Likewise, activation in the bilateral IFG did not differ between the high hormone phases and menses (all |*t*| < 2.03, all *p*_FDR_ > 0.35), but was significantly increased during the luteal compared to the pre-ovulatory phase (all *t* > 2.35, all *p*_FDR_ < 0.04). These effects were however not related to hormone levels (all *t* < 1.40, *p* > 0.16).

### Predicting performance by activation

To determine which brain areas most strongly predicted performance, we ran multiple regression models with performance as dependent variable and activation of the bilateral hippocampus, caudate and DLPFC as independent variables for both spatial navigation and verbal fluency (Table 1).

**Table 1:** Results of multiple regression model, predicting performance by brain activation

	Navigation Performance		Verbal fluency performance	
	*b* (SEb)	*T*	*b* (SEb)	*t*
HippocampusL	0.06 (0.08)	0.80	**0.28** (**0.10)**	2.60**
HippocampusR	**0.31** (**0.08)**	3.85***	−0.14 (0.11)	−1.34
CaudateL	−0.01 (0.09)	−0.08	0.13 (0.15)	0.83
CaudateR	**−0.20** (**0.10)**	−2.08*	−0.22 (0.16)	−1.35
DLPFC_L	**−0.15** (**0.05)**	−2.63**	**0.22** (**0.08)**	2.67**
DLPFC_R	−0.03 (0.05)	−0.47	−0.06 (0.08)	−0.77

*L* left, *R* right

*p < 0.05, **p < 0.01, ***p < 0.001

Significant predictors are highlighted in bold font

The strongest predictor of performance in the navigation task was activation of the right hippocampus, while the strongest predictor of performance in the verbal fluency task was activation of the left hippocampus. The stronger the hippocampal activation, the better was performance in these tasks. Furthermore, in both tasks activation of the left DLPFC related significantly to performance. Higher DLPFC activation related to better performance during verbal fluency, but worse performance during navigation. Furthermore, in both tasks activation of the right caudate related negatively to task performance, but the effect only reached significance for navigation.

### Fronto-frontal inter-hemispheric connectivity

**Fig. 3:**
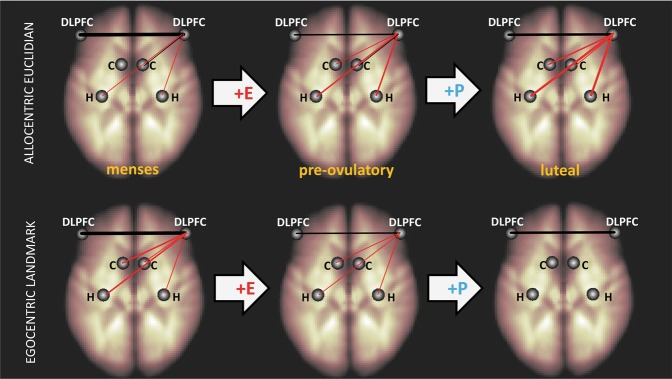
Menstrual cycle modulation of connectivity patterns in the navigation task. Connectivity between the left and right DLPFC decreased from menses to pre-ovulatory phase and increased from pre-ovulatory to luteal phase. Connectivity between right DLPFC and subcortical areas changed from menses to luteal phase, dependent on navigation strategy. Connectivity to the left hippocampus and caudate became more negative during Euclidian, but less negative during landmark-based navigation. Connectivity to the right hippocampus became more negative during allocentric, but less negative during egocentric navigation. Black connections: positive connectivity; red connections: negative connectivity. DLPFC dorsolateral prefrontal cortex, H hippocampus, C caudate. E estradiol, P progesterone. Thickness of lines represents connectivity strength

**Fig. 4:**
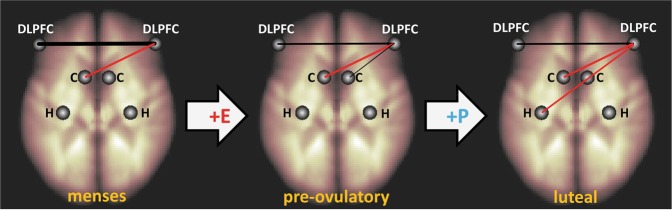
Menstrual cycle modulation of connectivity patterns in the verbal fluency task. Connectivity between the left and right DLPFC decreased during the pre-ovulatory phase. Connectivity between the right DLPFC and the left hippocampus decreased during the luteal phase. Black connections: positive connectivity; red connections: negative connectivity. DLPFC dorsolateral prefrontal cortex, H hippocampus, C caudate. E estradiol, P progesterone. Thickness of lines represents connectivity strength

Based on activation results, we predicted, that fronto-frontal connectivity would increase during the luteal phase. In both tasks, inter-hemispheric connectivity between the left and right DLPFC decreased significantly during the pre-ovulatory phase (navigation: *b* = −0.24, SE_b_ = 0.10, *t*_(391)_ = −2.39, *p* = 0.02, Fig. 3; verbal: *b* = −0.28, SE_b_ = 0.14, *t*_(171)_ = −2.04, *p* = 0.04, Fig. 4), and—in the case of the navigation task—increased significantly from pre-ovulatory to luteal phase (navigation: *b* = 0.21, SE_b_ = 0.10, *t*_(248)_ = 2.10, *p* = 0.04; verbal: *b* = 0.11, SE_b_ = 0.13, *t*_(102)_ = 0.82, *p* = 0.42). No differences were observed between menses and luteal phase (navigation: *b* = −0.05, SE_b_ = 0.10, *t*_(391)_ = −0.45, *p* = 0.65; verbal: *b* = −0.18, SE_b_ = 0.14, *t*_(171)_ = 1.31, *p* = 0.19).

### Fronto-subcortical connectivity

To further explore the role of the right DLPFC, connectivity between the right DLPFC and subcortical areas was extracted from a ROI-to-ROI analysis in both tasks.

#### Navigation

Overall, the right DLPFC was significantly negatively connected to the bilateral hippocampi (both *β* < −0.26, both *T* < −4.60, both *p*_FDR_ < 0.001) and the left caudate (*β* = −0.17, *T* = −3.54, *p*_FDR_ = 0.003), but not the right Caudate (*β* = 0.05, *T* = 0.95, *p*_FDR_ = 0.64). For no subcortical area, connectivity to the right DLPFC was modulated by a main effect of cycle phase and there were no changes in strategy- and perspective effects from menses to pre-ovulatory phase or from pre-ovulatory to luteal phase (all |*b*| < 0.56, all |*t*| < 1.94, all *p*_FDR_ > 0.20). However, from menses to luteal phase, significant cycle*strategy interactions were observed for the bilateral caudate and the left hippocampus (all *b* > 0.69, all SE_b_ < 0.32, *t*_(384)_ > 2.17, all *p*_FDR_ < 0.04) and a significant cycle*perspective interaction for the right hippocampus (*b* = 0.87, SE_b_ = 0.30, *t*_(384_) = 2.86, *p*_FDR_ = 0.02). From menses to luteal phase, connectivity between subcortical areas and the right DLPFC became more negative with the Euclidian strategy, but less negative with the landmark strategy (Fig. 3). From menses to luteal phase, connectivity between the right hippocampus and the right DLPFC became more negative during allocentric navigation, but less negative during ego-centric navigation (Fig. 3).

#### Verbal fluency

Overall, the right DLPFC was significantly negatively connected to the left caudate (*β* = −0.08, *T* = −3.25, *p*_FDR_ = 0.01), but not to the right caudate or the hippocampi (all |*β*| < 0.07, all |*T*| < 2.45, all *p*_FDR_ > 0.09). No cycle effect was observed in connectivity to the right hippocampus or left caudate (all |*b*| < 0.29, all |*t*| < 1.19, all *p* > 0.23). Connectivity between the right DLPFC and the left hippocampus or right caudate showed no differences between the high hormone phases and menses (all |*b*| < 0.28, all |*t*| < 2.09, all *p*_FDR_ > 0.12), but decreased significantly from pre-ovulatory to luteal phase irrespective of condition (both *b* < −0.37, both SE_b_ < 0.17, both *t*_(102)_ < −2.34, both *p*_FDR_ < 0.05; Fig. 4).

## Discussion

The present study set out to investigate menstrual cycle effects on brain activation in two different cognitive tasks, focusing on areas that emerged as targets for sex hormone actions in animal and human studies. Previous menstrual cycle research has often hypothesised opposite effects of sex hormones on typically “male-dominated” spatial and typically “female-dominated” verbal domains. Thus, the most striking finding of the present study is the very similar pattern of menstrual cycle effects on brain activation during spatial navigation and verbal fluency. Furthermore, in both tasks menstrual cycle changes in brain activation occur irrespective of cognitive strategy, suggesting that these changes are not a result of strategy shifts.

In both tasks, hippocampal activation is elevated during the pre-ovulatory phase and—in the left hemisphere—drops during the luteal phase, suggesting opposite effects of estradiol and progesterone on brain activation in this area. Indeed in both tasks an interactive effect of estradiol and progesterone on hippocampal activation could be confirmed, suggesting a stronger impact of estradiol in the presence of low progesterone levels. This is in line with findings from the animal literature reporting increased spine density in the hippocampus in the presence of high estradiol levels [29, 39], as well as findings from human structural MRI studies reporting increased grey matter volumes in the hippocampus in the pre-ovulatory phase of the menstrual cycle [31, 34].

In both tasks, hippocampal activation is positively correlated to performance. In line with previous literature, the right hippocampus emerged as the strongest predictor of performance during navigation [49, 50], while the left hippocampus emerged as the strongest predictor during verbal fluency [51, 52]. This positive association between hippocampal activation and performance, would suggest and improvement of performance during the pre-ovulatory phase, when estradiol levels and hippocampal activation peak. However, the slight improvement of performance in the pre-ovulatory phase did not reach significance after controlling for learning effects. Most likely, these non-significant findings in performance are attributable to limitations in the behavioural measures (see below). Another interpretation is that women are able to compensate for the luteal drop in hippocampal activation via other mechanisms.

The current results suggest that an increased activation of the caudate and right DLPFC might play a role in that respect. In both tasks these areas substantially increase their activation during the luteal phase and for navigation, a relationship to progesterone could also be confirmed. The right DLPFC already emerged as a target of luteal changes in a verbal working memory task [unpublished data], but also in previous fMRI-studies on spatial and verbal tasks [12, 16]. During a verbal n-back task [unpublished data], mental rotation [12] and by trend also verbal memory [16] activation of the right DLPFC increased in the luteal phase. The fact that the left DLPFC is the main predictor of performance but unresponsive to menstrual cycle changes, while activation of the right DLPFC increases in the luteal phase over a range of tasks, supports the idea of a compensatory role of this area. To test this idea, a series of connectivity analyses were performed.

In a first step, we tested for changes in inter-hemispheric connectivity along the menstrual cycle. If an increased connectivity between the left and right DLPFC was observed during the luteal phase, the increased activation of the right DLPFC could be interpreted as supporting the left DLPFC to uphold task-performance. However, instead of an increase in inter-hemispheric connectivity during the luteal phase, we observed a decrease during the pre-ovulatory phase that was only partly reversed during the luteal phase. It can be speculated that the increased activation of the right DLPFC during the luteal phase reflects an attempt to reconnect to its left-hemispheric counterpart. It is also possible that the decoupling of the right DLPFC from the left DLPFC frees the right DLPFC for other tasks. Accordingly, the decoupling itself may be compensatory, as the right DLPFC reduces its role as supporter of the left DLPFC and becomes a more independent player in the network supporting task performance. Menstrual cycle-dependent changes in inter-hemispheric connectivity have repeatedly been described [53, 54].

To explore this independent role of the right DLPFC further, a second step in the connectivity analyses was to test for connectivity changes between the right DLPFC and subcortical areas. In the verbal fluency task, connectivity to the left hippocampus—an area driving performance during this task—was significantly decreased, i.e. became more negative during the luteal phase. This alteration suggests that the up-regulation of right DLPFC activation during the luteal phase is involved in the simultaneous down-regulation of hippocampal activation.

A more complex pattern emerged in the navigation task. While performance and brain activation in both tasks did not yield any shifts in cognitive strategy along the menstrual cycle, such shifts were reflected in connectivity of the right DLPFC to subcortical areas in the navigation task. In the navigation task, connectivity of the right DLPFC to the hippocampi was modulated by perspective and strategy. The negative connectivity between the right DLPFC and subcortical areas was decreased during egocentric/landmark-based navigation, but increased during allocentric/Euclidian navigation. This nicely parallels previous results that women show improved performance in the luteal phase, when instructions are phrased from an egocentric perspective and using a landmark-based strategy [21]. Accordingly, if negative connectivity is interpreted as inhibitory control of frontal areas over subcortical areas, women in the current sample show a decrease in top-down inhibition during the luteal phase in precisely those conditions for which women of previous studies show improved performance during the luteal phase. It is an interesting observation that navigation strategies affect menstrual cycle changes in connectivity measures, while menstrual cycle changes in brain activation occur irrespective of cognitive strategy. This suggests that strategy shifts depend on the synchronicity of activity between cortical and subcortical areas irrespective of the strength of this activity. Albeit speculatively, such a scenario is possible, if the increased BOLD-response in the right DLPFC supports a shift in the frequency of oscillatory activity at the neural level. Such a frequency shift may not only explain the general decoupling from its left-hemispheric counterpart, but also the strategy-dependent connectivity changes to subcortical areas, provided that the frequency of neural activity in subcortical areas does not change along the menstrual, but differs between task conditions. In line with this idea, multiple EEG/MEG studies suggest a shift in frontal alpha asymmetry along the menstrual cycle with stronger alpha activity in right-frontal areas during the mid-luteal phase [55, 56].

The fact that no performance changes (also no strategy-changes) along the menstrual cycle are observed in the present study is likely attributable to two issues: (i) the less sensitive performance measure employed during the present study and (ii) the learning effect that inadvertedly occurs in a repeated measures design. In the previous behavioural version of the navigation task [38], participants received three lines of directions, describing a path to a target location through the environment. Their task was to reach the target location as fast as possible and performance was reflected in the time they needed to complete the task. However, navigation times differed substantially between participants, which represents a potential confound for brain activation. Accordingly in the present study, each item was presented for a fixed time of 30 s and participants had to reach as many targets as possible. Similarly, in the verbal fluency task, a covert version was used to avoid movement artefacts, resulting in only a rough estimate (button presses) of performance. Thus, the tasks in the present study were optimised for assessing changes in brain activation and connectivity, while previous behavioural studies were optimized for assessing even subtle differences in performance. Accordingly, well-planned behavioural studies using more elaborate versions of these tasks [as e.g. employed in [21, 38]] and including three or more cycle phases are much needed to explore the behavioural implications of the current study. Furthermore, the field would profit from a healthy mix of within-subjects like the current one and sufficiently-powered between-subjects studies. While within-subjects studies avoid confounds by group differences in e.g. IQ, social status, education or relationship status, learning effects may overshadow subtle menstrual cycle changes.

In summary our results suggest that irrespective of the task, estradiol boosts hippocampal activation during the pre-ovulatory phase and progesterone boost right-hemispheric fronto-striatal activation during the luteal phase. Connectivity analyses suggest that the increase in right-hemispheric frontal activation is the result of inter-hemispheric decoupling and is involved in the down-regulation of hippocampal activation on the one hand and the modulation of cognitive strategies on the other hand.

## Funding and disclosure

The authors declare no conflict of interest or competing interests. This study was funded by the Austrian Science Fund (P28261, W1233-G17).

## Supplementary information

Supplementary Material

Supplementary Table
