# Supplementary material for: The cycling brain: menstrual cycle related fluctuations in hippocampal and fronto-striatal activation and connectivity during cognitive tasks
Source: Neuropsychopharmacology. 2019 Jun 13;44(11):1867–75. doi: 10.1038/s41386-019-0435-3 (PMC6785086; doi:10.1038/s41386-019-0435-3)
Supplement: Supplementary file 1 — Supplementary Material [file 41386_2019_435_MOESM1_ESM.docx]

**Effects of session and strategy on task performance, brain activation and connectivity**

**Performance:**

Navigation: Performance increased significantly with the number of test sessions (b = 0.33, SE_b_ = 0.02, t_(391)_ = 13.76, p < 0.001). Participants reached significantly more targets with egocentric compared to allocentric directions (b = 0.87, SE_b_ = 0.05, t_(391)_ = 18.97, p < 0.001) and with landmark based instructions compared to Euclidian instructions (b = 0.13, SE_b_ = 0.05, t_(391)_ = 2.88, p = 0.004).

Verbal Fluency: Performance increased significantly with the number of test sessions (b = 0.09, SE_b_ = 0.04, t_(171)_ = 2.49, p = 0.01). Participants were able to produce significantly more words with the clustering instruction compared to the switching instruction (b = -0.51, SE_b_ = 0.07, t_(171)_ = -7.23, p < 0.001).

**Brain activation:**

Navigation: Activation in the bilateral hippocampus, parahippocampus, and putamen (all b > 0.07, all t > 2.07, all p_FDR_ < 0.05) increased with session, while activation in the bilateral caudate and right DLPFC decreased with session (all |b| > 0.10, all |t| > 2.50, all p_FDR_ < 0.02). Perspective and strategy did not affect activation in the hippocampus, putamen or caudate (all |b| < 0.14, all |t| < 2.20, all p_FDR_ > 0.06), but the bilateral parahippocampus showed stronger activation for egocentric compared to allocentric directions (right: b = 0.18, SE_b_ = 0.06, t = 2.90, p_FDR_ = 0.02; but left: b = 0.12, SE_b_ = 0.06, t = 1.85, p_FDR_ = 0.12) and for landmark-based compared to Euclidian directions (both b > 0.46, both SE_b_ > 0.06, both t > 7.46, both p_FDR_ > 0.001). Furthermore, the bilateral DLPFC showed stronger activation for allocentric compared to egocentric directions (both |b| > 0.14, both |t| > 2.20, both p < 0.04), and for landmark-based compared to Euclidian instructions (left: b = 0.18, SE_b_ = 0.07, t = 2.74, p_FDR_ = 0.02; but right: b = -0.02, SE_b_ = 0.07, t = -0.23, p _FDR_ = 0.90).

Verbal Fluency: Session did not affect activation in any area (all |b| < 0.08, all |t| < 1.71, all p_FDR_ > 0.23), except for the left caudate (b = -0.14, SE_b_ = 0.04, t_(171)_ = -3.39, p_FDR_ = 0.009). Condition (clustering vs. switching) did not affect activation any area (all |b| < 0.21, all |t| < 2.42, all p_FDR_ > 0.05), except the bilateral caudate (left: b = -0.31, SE_b_ = 0.08, t_(171)_ = -3.77, p_FDR_ = 0.002; right: b = -0.23, SE_b_ = 0.08, t_(171)_ = -.94, p_FDR_ = 0.02).

**Brain connectivity:**

Navigation: Inter-hemispheric connectivity increased significantly with the number of test sessions (b = 0.09, SE_b_ = 0.04, t_(391)_ = 2.22, p = 0.03), while fronto-subcortical connectivity did not change with the number of test sessions (all |b| < 0.08, all |t| < 1.78, all p_FDR_ > 0.32). Perspective did not affect inter-hemispheric or fronto-subcortical connectivity (all |b| < 0.35, all |t| < 1.70, all p_FDR_ > 0.27) with the exception of connectivity between the right DLPFC and the right hippocampus, which was stronger for allocentric compared to egocentric directions (b = -0.59, SE_b_ = 0.21, t = -2.75, p_FDR_ = 0.03). Strategy did not affect inter-hemispheric or fronto-subcortical connectivity to any area (all |b| < 0.47, all |t| < 2.08, all p_FDR_ > 0.15).

Verbal Fluency: Inter-hemispheric connectivity increased significantly with the number of test sessions (b = 0.13, SE_b_ = 0.06, t_(171)_ = 2.28, p = 0.02), but was not affected by condition (b = 0.07, SE_b_ = 0.11, t_(171)_ = 0.63, p = 0.53). Session and condition did not affect fronto-subcortical connectivity (all |b| < 0.15, all |t| < 1.17, all p > 0.24).
