# Supplementary material for: The cycling brain: menstrual cycle related fluctuations in hippocampal and fronto-striatal activation and connectivity during cognitive tasks
Source: Neuropsychopharmacology. 2019 Jun 13;44(11):1867–75. doi: 10.1038/s41386-019-0435-3 (PMC6785086; doi:10.1038/s41386-019-0435-3)
Supplement: Supplementary file 2 — Supplementary Table [file 41386_2019_435_MOESM2_ESM.docx]

|  | **navigation** | | | | | | **verbal fluency** | | | | | |
| --- | --- | --- | --- | --- | --- | --- | --- | --- | --- | --- | --- | --- |
|  | F > M | L > M | L > F | E | P | E*P | F > M | L > M | L > F | E | P | E*P |
| **subcortical** |  |  |  |  |  |  |  |  |  |  |  |  |
| HippocampusL | **0.25^**^ (0.08)** | -0.03 (0.08) | **-0.26^**^ (0.08)** | **0.18^*^ (0.06)** | -0.01 (0.05) | **-0.11^**^ (0.04)** | 0.10 (0.12) | -0.16 (0.12) | **-0.25^*^ (0.11)** | 0.21 (0.08) | -0.07 (0.06) | **-0.16^*^ (0.05)** |
| HippocampusR | **0.22^*^ (0.08)** | **0.20^*^ (0.08)** | -0.02 (0.07) | **0.22^**^ (0.06)** | 0.06 (0.05) | **-0.19^***^ (0.04)** | 0.04 (0.14) | -0.09 (0.13) | -0.12 (0.12) | 0.16 (0.09) | 0.0004 (0.07) | **-0.17^*^ (0.05)** |
| PutamenL | 0.07 (0.09) | 0.09 (0.09) | 0.01 (0.09) |  |  |  | -0.11 (0.12) | -0.03 (0.12) | 0.07 (0.12) |  |  |  |
| PutamenR | 0.13 (0.08) | 0.14 (0.08) | -0.01 (0.08) |  |  |  | -0.11 (0.13) | -0.04 (0.13) | 0.06 (0.12) |  |  |  |
| CaudateL | **0.24^**^ (0.08)** | **0.34^***^ (0.08)** | 0.06 (0.08) | 0.11 (0.06) | 0.06 (0.04) | **-0.12^**^ (0.03)** | -0.003 (0.10) | **0.31^*^ (0.10)** | **0.30^**^ (0.09)** | -0.01 (0.08) | 0.08 (0.05) | -0.005 (0.04) |
| CaudateR | 0.16 (0.08) | **0.41^***^ (0.08)** | **0.22^*^ (0.07)** | 0.08 (0.06) | **0.14^**^ (0.04)** | **-0.14^***^ (0.03)** | -0.08 (0.10) | **0.28^*^ (0.10)** | **0.36^***^ (0.09)** | -0.02 (0.08) | 0.08 (0.06) | -0.01 (0.04) |
| **cortical** |  |  |  |  |  |  |  |  |  |  |  |  |
| DLPFC_L (BA 46) | 0.001 (0.08) | 0.03 (0.08) | 0.02 (0.08) |  |  |  | -0.01 (0.09) | 0.02 (0.09) | 0.02 (0.10) |  |  |  |
| DLPFC_R (BA 46) | 0.13 (0.08) | **0.31^***^ (0.08)** | 0.15 (0.07) | 0.06 (0.06) | **0.16^**^ (0.05)** | **-0.11^**^ (0.04)** | -0.09 (0.08) | 0.16 (0.08) | **0.24^*^ (0.08)** | -0.07 (0.06) | 0.07 (0.05) | 0.02 (0.04) |
| ParahippocampusL | **0.43^***^**  **(0.08)** | **0.20^*^ (0.08)** | **-0.21^*^ (0.08)** | **0.15^*^ (0.06)** | 0.003 (0.05) | **-0.09^**^ (0.03)** |  |  |  |  |  |  |
| ParahippocampusR | **0.20^*^ (0.08)** | **0.26^**^ (0.08)** | 0.06 (0.08) | 0.02 (0.06) | 0.05 (0.04) | -0.01  (0.03) |  |  |  |  |  |  |
| IFG_L (BA 44/45) |  |  |  |  |  |  | -0.10 (0.11) | 0.16 (0.11) | **0.25^*^ (0.11)** | -0.09 (0.08) | 0.05 (0.06) | -0.02 (0.05) |
| IFG_R (BA 44/45) |  |  |  |  |  |  | -0.25 (0.12) | 0.13 (0.12) | **0.36^*^ (0.12)** | -0.01 (0.09) | 0.06 (0.07) | 0.01 (0.05) |

**Supplementary Table 1: Effect sizes b (SE_b_) for cycle comparisons of brain activation.** M = menses, F = pre-ovulatory, L = luteal, E = estradiol, P = progesterone. L = left, R = right, BA = Brodman area. ^*^p_FDR_ < 0.05, ^**^p_FDR_ < 0.01, ^***^p_FDR_ < 0.001. Fields are displayed in grey, if analyses were not performed.
